# Evaluation of management practices in rice–wheat cropping system using multicriteria decision-making methods in conservation agriculture

**DOI:** 10.1038/s41598-024-58022-w

**Published:** 2024-04-13

**Authors:** Tufleuddin Biswas, Anurup Majumder, Shamik Dey, Anwesha Mandal, Soumik Ray, Promil Kapoor, Walid Emam, Sahely Kanthal, Alessio ISHIZAKA, Adelajda Matuka

**Affiliations:** 1 Department of Agricultural Economics and Statistics, Centurion University of Technology and Management (CUTM), Paralakhemundi, Odisha 761211 India; 2 Department of Agricultural Statistics, Bidhan Chandra Krishi Viswavidyalaya, Mohanpur, West Bengal 741252 India; 3 School of Agricultural Sciences, JIS University, Kolkata, West Bengal 700109 India; 4 School of Agricultural Sciences, G D Goenka University, Sohna Rural, Haryana India 122103; 5 Assistant Scientist, Plant Pathology, CCSHAU, Hisar, India 125004; 6 Department of Statistics and Operations Research, Faculty of Science, King Saud University, P.O. Box 2455, 11451 Riyadh, Saudi Arabia; 7 School of Agriculture, Swami Vivekananda University, Barrackpure, West Bengal 700121 India; 8 Department of Supply Chain and Decision Making, NEOMA Business School, Rouen, France 76130; 9 Department of Economics, University of Bologna, 40126 Bologna, Italy

**Keywords:** Agroecology, Ecological modelling

## Abstract

In this study, we employed two multiple criteria decision-making (MCDM) methods, namely the Technique for Order Preference by Similarity to Ideal Solution (TOPSIS) and the Analytic Hierarchic Process (AHP), to determine the best management choice for the cultivation of wheat with a regime of conservation agriculture (CA) practices. By combining alternative tillage approaches, such as reduced tillage and zero tillage, with the quantity of crop residues and fertilizer application, we were able to develop the regime of CA practices. The performance of the regimes compared to the conventional ones was then evaluated using conflicting parameters relating to energy use, economics, agronomy, plant protection, and soil science. TOPSIS assigned a grade to each alternative based on how close it was to the ideal solution and how far away it was from the negative ideal solution. However, employing AHP, we determined the weights of each of the main and sub-parameters used for this study using pairwise comparison. With TOPSIS, we found ZERO1 (0% residue + 100% NPK) followed by ZERO4 (50%residue + 100% NPK), and ZERO2 (100% residue + 50% NPK) were the best performing tillage-based alternatives. To best optimize the performance of wheat crops under various CA regimes, TOPSIS assisted the decision-makers in distinguishing the effects of the parameters on the outcome and identifying the potential for maneuvering the weak links. The outcomes of this investigation could be used to improve management techniques for wheat production with CA practices for upscaling among the farmers.

## Introduction

Agricultural sustainability now-a-days is paramount as it strives to optimize crop output while minimizing environmental impact. Conservation agriculture is such a system. It works through three principles, viz., less tillage, more soil cover, and improved rotations, and offers multifaceted benefits influencing different aspects of crop cultivation, including energy use, soil health, soil biodiversity, economics, etc^1,2^. Attempts are made to upscale the CA practices in the rice–wheat system, the foundation of food security of SE Asian countries**.** In such a system, rice needs to be planted first, followed by wheat, with minimal to no-tillage with and without crop residue of rice under CA. In fact, there are many variants of CA, viz., zero-tillage, reduced tillage, with and without crop residues, etc. The performance of such variants also varies hugely^3,4^.

Previous investigations on CA-based rice–wheat system exclusively assessed management approaches by examining productivity, soil, or protection factors in isolation, with a primary emphasis on economic considerations. The proper selection of efficient wheat crop management strategies is pivotal for attaining sustainable agricultural outcomes based on several criteria. In the present study, numerous decision-making parameters, including agronomic yield, soil health, crop protection, energy use, and economic factors, were selected, incorporating the opinions of experts from diverse agricultural disciplines. This research sheds light on the complex issues associated with wheat crop management within the framework of CA, employing decision-making methods.

The goal of the multiple criteria decision-making (MCDM) technique is to help in choosing the best option or options from a range of options^5^. This approach is commonly employed in optimizing the performance of diverse organizations, such as those operating in the industrial, management, and administrative domains, both within the public and private sectors^6–9^. The deliberate selection of MCDM methods, particularly the Analytic Hierarchy Process (AHP) and Technique for Order of Preference by Similarity to Ideal Solution (TOPSIS), demonstrates a conscious decision to address the intricacies involved in agricultural decision-making^10,11^. The AHP is well known for its capacity to manage intricate decision-making situations involving several criteria and options. Its pairwise comparison methodology makes it possible to evaluate criteria and options in an organized and systematic manner^12,13^. In contrast, TOPSIS works exceptionally well when there are conflicting criteria and both qualitative and quantitative elements are considered. It considers the proximity of each alternative to the ideal solution and far from the negative ideal solution (NIS)^14^. The use of the MCDM technique is not rare in the agricultural decision-making process^15–18^, but the performance of the conjoint use of TOPSIS and AHP is hardly known. Such use of multi-attribute decision-making methodologies is useful to tackle more complex problems with multiple conflicting parameters.

Numerous empirical investigations have used TOPSIS methodologies with weights derived from entropy theory^15,19–22^, frequently failing to take the accuracy of determining the weights of specific criteria into sufficient account. In order to overcome this problem, the present work used weights that were obtained from the AHP technique, taking advantage of its ability to handle complex decision-making situations by using methodical pairwise comparisons to establish criteria weights. Moreover, the inclusion of consistency checks improves the weight findings' transparency and dependability. Selecting suitable management techniques within the framework of conservation agriculture is imperative for specific agro-climatic regions. Consequently, the utilization of MCDM becomes crucial in determining the optimal management alternative within CA practices. In light of this, the current study used AHP and TOPSIS methodologies to determine the best management strategies for rice–wheat cropping systems under a regime of CA practices. The study contributes to the expanding body of research on conservation agriculture techniques while addressing the real-world obstacles that farmers and decision-makers need to conquer to put effective conservation measures into reality.

## Materials and methods

### Experimental details

During the winter/*Kharif* and summer/*Rabi* seasons of 2018–2019, an experiment was conducted at the Balindi Research Farm (22.96° N, 88.53° E), Bidhan Chandra Krishi Viswavidyalaya, West Bengal. Based on the intensity/degree of tillage (energy utilized) imposed, the field (2.0 ha) was divided into three tillage plots: Conventional Tillage (CONV), zero Tillage (ZERO), and Reduced Tillage (REDUC). Each tillage plot was subsequently divided into five sub-plots, each with a different combination of nutrients and residues.

To establish a fine tilt and homogeneous seedbed, a conventional tillage plot was prepared by performing primary tillage with a tractor-drawn disc plow, followed by two passes of a rigid-tyne cultivator and rotary tiller as secondary tillage. While implementing zero tillage, no soil disturbance was made, although sowing was done using a zero-till seed-cum-fertilizer drill. The plot for reduced tillage got sequential tillage operations with a single run of a wide-tyne cultivator and a single pass of an offset disc harrow as secondary tillage. The recommended dose of N, P and K fertilizer for each crop cultivation was 64, 48 and 32 kg per hectare, respectively, based on soil test values. Different proportion (50, 75 and 100%) of this recommended amount was combined with different proportion (0, 50 and 100% of the produced amount in the preceding rice) of rice residues, creating in total five combinations for each tillage treatment, as given in Table 1.

**Table 1:** Details of the fifteen alternatives (management practices).

Tillage	Nutrient-crop residue combination
Conventional tillage (CONV)	CONV1: 0% residue + 100% NPK
	CONV2: 100% residue + 50% NPK
	CONV3: 100% residue + 75% NPK
	CONV4: 50% residue + 100% NPK
	CONV5: 50% residue + 75% NPK
Reduced tillage (REDUC)	REDUC1: 0% residue + 100% NPK
	REDUC2: 100% residue + 50% NPK
	REDUC3: 100% residue + 75% NPK
	REDUC4: 50% residue + 100% NPK
	REDUC5: 50% residue + 75% NPK
Zero tillage (ZERO)	ZERO1: 0% residue + 100% NPK
	ZERO2: 100% residue + 50% NPK
	ZERO3:100% residue + 75% NPK
	ZERO4: 50% residue + 100% NPK
	ZERO5: 50% residue + 75% NPK

### Database

#### The background of the selection of various decision-making criteria

A database was created for different parameters relating to soil, agronomy, energy use, plant protection (agricultural entomology and plant pathology), and economics during the cultivation of the crop (Table2) by consulting with different agriculture experts (Supplementary material, S1). The parameters chosen were linked with the three principles of CA and related ecosystem services of soil which influenced the growth of the crop.

**Table 2:** Various main and sub-parameters along with details of the preference functions used in the TOPSIS method.

Relative importance of the main parameter (criterion)	Sub-criteria at each level of the main criterion	Preferred function
Agronomic	Grain yield (kg ha^−1^)	Maximum
Soil	Soil organic carbon (g kg^−1^)	Maximum (except bulk density: minimum)
	Nitrogen (kg ha^−1^)	
	Phosphorus (kg ha^−1^)	
	Potassium (kg ha^−1^)	
	Zinc (mg kg^−1^)	
	Sulphur (mg kg^−1^)	
	MBC (mg C kg^−1^ soil)	
	Dehydrogenase (µg TPFg^−1^ h^−1^)	
	Bulk density (Mg m^−3^)	
	WHC (%)	
Energy use	Human (MJ ha^−1^)	Minimum
	Chemical (MJ ha^−1^)	
	Electricity (MJ ha^−1^)	
	Fertilizer (MJ ha^−1^)	
	Fuel (MJ ha^−1^)	
	Machinery (MJ ha^−1^)	
	Irrigation (MJ ha^−1^)	
	Residue (MJ ha^−1^)	Maximum
Plant protection	Soil mites (per 100 gm soil)	Maximum
	Protura (per 100 gm soil)	
	Collembola (per 100 gm soil)	
	Spider (per 100 gm soil)	
	Disease severity (%)	Minimum
	Disease incidence (%)	
Economic	Benefit–cost ratio	Maximum

#### Agronomic criteria

A crucial indicator of agricultural productivity is yield, and wheat grain yield was recorded.

#### Soil criteria

The three principles of CA employed in various intensities are known to influence soil health. To assess the health, a number of physicals (2), chemical (6) and biological (2) attributes were analyzed (Table 2) for soils under all those fifteen different intensities of CA practices collected after harvesting the crop and used as soil criteria for identifying the best alternatives.

#### Energy criteria

Modern agriculture has become energy intensive. It affects the cost of production. A scientific evaluation is, therefore, required to reduce input energy used in land preparation, fertilizers, agrochemicals, and irrigation to promote energy efficiency while using conservation agriculture, including its different components like zero tillage, reduced tillage, crop residue retention and use of specialized farm machinery, etc. A few relevant parameters of energy components were, therefore, recorded in this study as criteria for choosing the best alternatives.

#### Plant protection criteria

Soil mesofauna plays a crucial role in the functioning of soil ecosystem by breaking down organic matter and augmenting soil fertility. CA intensities have a significant influence on the diversity and richness of those mesofauna. Again, among the soil microarthropods, soil mites represent the most diverse and numerically abundant group in the soil ecosystem with diverse feeding biology^23^. Minimum tillage is also known to augment and accelerate the emergence of more predators and parasitoids than plowed plots^24^. Keeping these in mind, a number of these organisms that commonly occur in the region were also enumerated in soils under all the tested alternatives (Table 2) for identifying the best.

#### Economic criteria

Cultivation of crops under conservation agricultural practices helped in the optimization of resource use and maximization of crop productivity; ultimately, it led to an increase in the benefit–cost ratio.

Selection of all these parameters was done based on the opinions of experts belonging to different disciplines with previous research experiences in CA to choose the best criteria for such assessment.

**Figure 1:**
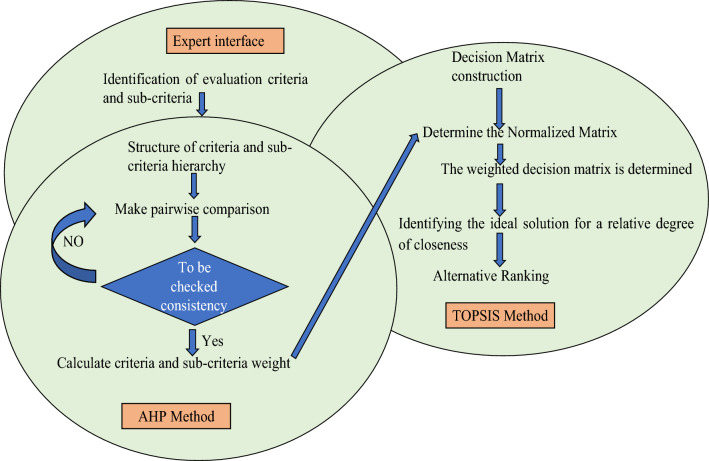
Schematic overview of the AHP and TOPSIS procedures.

With these background criteria, two MCDM techniques, viz., TOPSIS and AHP, were used for identifying the best alternatives out of the fifteen tested because of their edges over the other methods for such assessment (Fig. 1).

### TOPSIS procedure

Suppose there are K alternatives, namely S_1_, S_2_… S_K_ and N parameters (criteria) to evaluate each alternative S_k_ denoted as C_1_, C_2_… C_N_ (as shown in Table 1). The value of the kth alternative on the nth criteria is represented as ${X}_{k\times n}$. This denotes the specific value or measurement associated with the kth alternative and the nth criterion. It may be noted that ${S}_{k}=\left({x}_{k1}, {x}_{k2}, \dots {x}_{kN}\right)$ and ${C}_{n}=\left({x}_{1n}, {x}_{2n}, \dots {x}_{nk}\dots {x}_{Kn}\right)$; k represents the range of alternatives, ranging from 1 to K, while n represents the range of criteria, ranging from 1 to N. These values allow for the systematic evaluation and comparison of each alternative across all criteria.

#### Initial data matrix

The TOPSIS method's^14^ basic data structure, which considers alternatives ordered row-by-row and parameters structured column-by-column, is displayed in the initial data Matrix (Table 3)

$$({\mathbf{X}}_{kn} )_{K \times N} = \left[ {\begin{array}{*{20}c} {x_{11} } & {...} & {x_{1N} } \\ \vdots & {x_{kn} } & \vdots \\ {x_{K1} } & \cdots & {x_{KN} } \\ \end{array} } \right]$$

**Table 3:** Data matrix.

Criterion/Alternatives	C_1_	C_2_	…	C_n_	…	C_N_
S_1_	${x}_{11}$	${x}_{12}$	…	${x}_{1n}$	…	${x}_{1N}$
S_2_	${x}_{21}$	${x}_{22}$	…	${x}_{2n}$	…	${x}_{2N}$
…	…	…	…		…	…
S_k_	${x}_{k1}$	${x}_{k2}$	…	${x}_{kn}$	…	${x}_{kN}$
…	…	…	…		…	…
S_K_	${x}_{K1}$	${x}_{K2}$	…	${x}_{Kn}$	…	${x}_{KN}$

The given number ${x}_{kn}$ and their respective matrix.

##### Determine the normalized matrix

The nth criteria vector Cn is normalized as TCn. When

1 $${{\text{TC}}}_{{\text{n}}}=\frac{{C}_{n}}{\left|{C}_{n}\right|}= ({{\text{x}}}_{1n} /|{C}_{n} |,{{\text{x}}}_{2n}/|{C}_{n} |\dots {{\text{x}}}_{kn} /|{C}_{n}|)$$

where $|{C}_{n} |= \sqrt{\sum_{k=1}^{K}{\left({x}_{kn}\right)}^{2}}$, is the Euclidian length or norm of ${C}_{n}$, the new criteria vectors are unit-free and directly comparable because they have the same length.

##### Calculating the selected criteria weights using AHP

**Figure 2:**
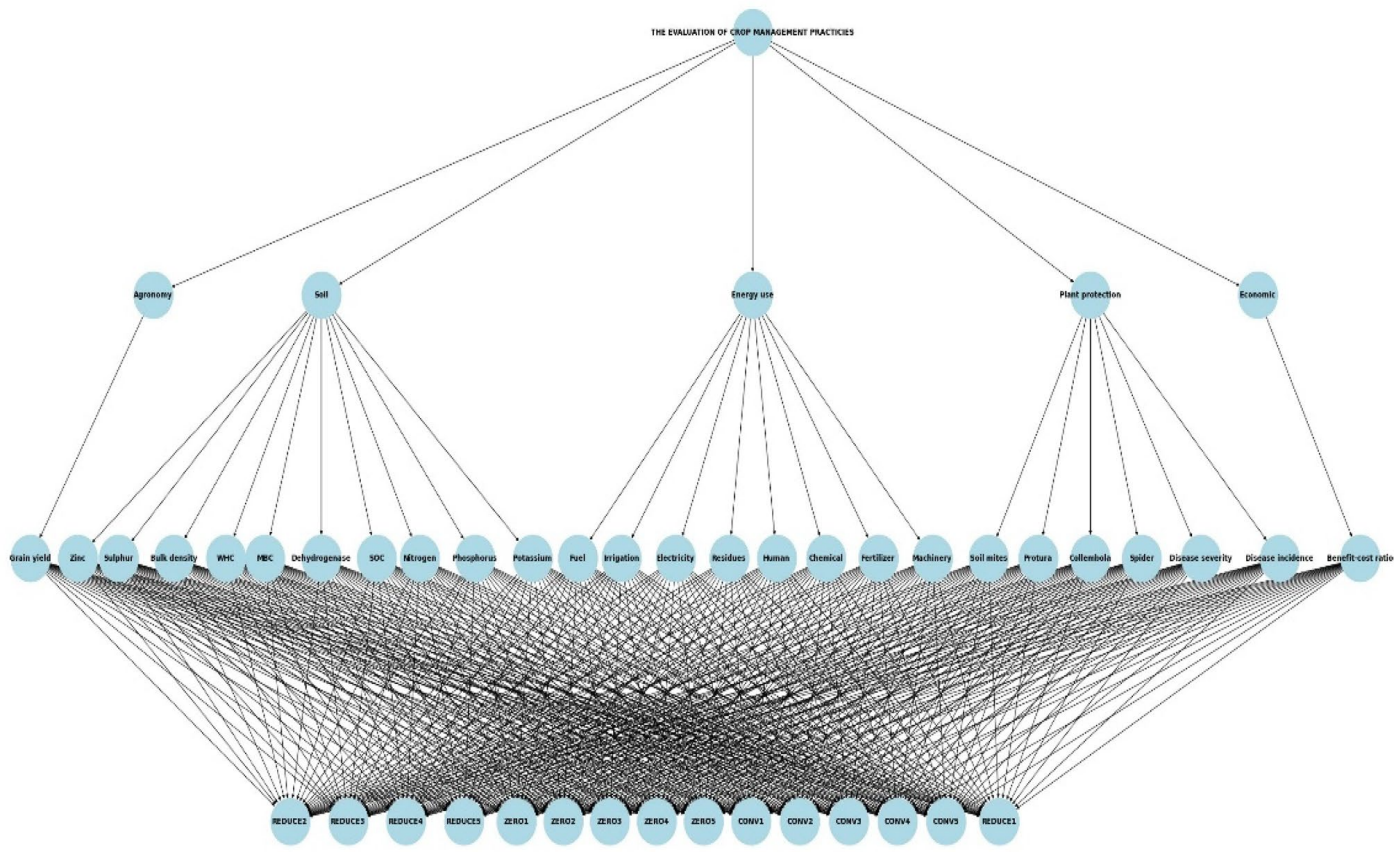
Structure of AHP tree produced in Python 3.12 (https://www.python.org/); Matplotlib package (https://matplotlib.org/).

According to the AHP method^25^, the weights of the chosen criteria were determined in the pairwise comparison matrix in four steps, which were (I) Develop a model for the business, (II) Derive priorities (weight) for the criteria, (III) Consistency check (weight assigned correctly or not), (IV) Derive overall or global priorities (model synthesis) and final decision. A relevant management practice assessment can be conducted by using pairwise comparison analysis to assign varying degrees of weight to the criteria^26^. The rankings were established using particular criteria in a pairwise comparison matrix, and they were subsequently compared based on the opinions of multiple experts in wheat production from various backgrounds. In order to obtain expert opinions, questionnaires were created (Supplementary materials, S2–S5). Python 3.12 (https://www.python.org/), in conjunction with the Matplotlib package (https://matplotlib.org/), was employed to generate the AHP tree shown in Fig. 2. This tree encompasses criteria, sub-criteria, and alternatives.

The pairwise comparison matrix is now applied to compare a collection of n criteria pairwise based on their relative relevance weights (Table 4).

$${\mathbf{A = }}\left[ \begin{gathered} {\text{a}}_{{{11}}} \,{\text{a}}_{{{12}}} \,...\,{\text{a}}_{{{\text{1j}}}} ...\,\,{\text{a}}_{{{\text{1n}}}} \hfill \\ {\text{a}}_{{{21}}} \,{\text{a}}_{{{22}\,}} \,...\,{\text{a}}_{{{\text{2i}}}} ...\,\,{\text{a}}_{{{\text{2n}}}} \hfill \\ ....\,\,....\,\,\,....\,\,....\,\,.... \hfill \\ {\text{a}}_{{{\text{i1}}}} \,{\text{a}}_{{{\text{i2}}\,\,}} \,...\,{\text{a}}_{{{\text{ij}}}} \,...\,\,{\text{a}}_{{{\text{in}}}} \hfill \\ {\text{a}}_{{{\text{1n}}}} \,{\text{a}}_{{{\text{2n}}}} \,...\,{\text{a}}_{{{\text{in}}}} \,...\,{\text{a}}_{{{\text{nn}}}} \hfill \\ \end{gathered} \right]\,,\,a_{ii} = 1,\,a_{ij} \, = \frac{1}{{a_{ji} }},\,a_{ji} \ne 0\,$$

**Table 4:** The fundamental scales of absolute numbers used for pair-wise comparison.

Intensity of importance	Definition	Explanation
1	Same Importance	When, both activities equally contribute to the objective
2	Weak or slight	
3	Fairly significant	One activity is slightly preferred or given a slight advantage over the other centered on skill and verdict
4	Slightly higher than moderate importance	
5	Significant importance	Based on knowledge and decision, one action is significantly favored over the other
6	Strong plus	
7	Exhibited a pronounced importance	One action demonstrates strong dominance over another, clearly showcased in practical implementation
8	Extremely potent	
9	Utmost significance	The demonstration supporting the encouragement of one action over another is of the utmost level of confirmation
Reciprocals of above	If activity i is assigned a non-zero value in comparison to activity j, then activity j is assigned the reciprocal value in comparison to i	A statement that is logical and reasonable
1.1–1.9	In the case of activities being in close proximity to each other	While it may be challenging to determine the exact value when comparing contrasting activities, the relatively small differences in numbers might not be easily noticeable. However, these subtle variations can still indicate the relative importance of the activities

##### Checking consistency index

If the number of comparisons, given by n(n − 1)/2, aligns with the number of criteria, denoted as n, then the elements {a_ij_} will adhere to the following conditions: w_i_/w_j_ = 1/a_ji_ and a_ii_ = 1, where i is from 1 to m and j is from 1 to n. The parameters are denoted by a_1_, a_2_….a_n_. Finding the eigenvector (w) that fulfills the conditions Aw = ${\tau }_{max}w$ and ${\tau }_{max}\ge n$ with respective ${\tau }_{max}$, where ${\tau }_{max}$ is the biggest eigenvalue of matrix **A**, yields the comparative weights. The discrepancy, if any, between ${\tau }_{max}$ and n is a sign that the judgments are inconsistent. The judgments are proven to be consistent if the ${\tau }_{max}=n$. The last step is to generate a Consistency Index (CI) using the formula. $\frac{({\tau }_{max}-n)}{(n-1)}$. With the help of their Consistency Indices, Saaty created a sizable sample of random matrices in increasing order, which would be compared against judgments made entirely at random. The consistency ratio (CR) is represented by the ratio of the consistency index and the index for the related random matrix table. Saaty said that if the ratio is higher than 0.1, the judgments may be too erratic to be trusted. In general, a CR value of 0.1 or less can be accepted, and a CR value of zero denotes that the judgment is completely consistent^25^. Thus, from the AHP methods, the weight of the criteria (W_n_) is: ${{\varvec{W}}}_{{\varvec{n}}}{=({w}_{1},{w}_{2},\dots .{w}_{n})}{\prime}$ and $\sum_{n=1}^{N}{W}_{n}=1$

##### The weighted decision matrix is determined:

The resulting normalized decision matrix's columns are then multiplied by the related weights, ${W}_{n}$, found in Eq. (2). Additionally, the weighted and normalized decision matrix is derived from the formula,

2 $${V}_{kn}={{\text{TC}}}_{{\text{n}}}{W}_{n}$$

##### Finding the ideal solution

The positive ideal solution is formed by selecting the best value for each attribute from the weighted decision matrix, as illustrated in Eq. (3). Conversely, the negative ideal solution is constructed using the worst value for each attribute from the weighted decision matrix, as illustrated in Eq. (4).

3 $$S^{ + } = (S_{1}^{ + } ,S_{2}^{ + } , \ldots ..S_{K}^{ + } )$$

4 $$S^{ - } = (S_{1}^{ - } ,S_{2}^{ - } , \ldots ..S_{K}^{ - } )$$

Now, the ideal value and negative ideal value are determined by

$${S}_{k}^{+}=\{Max{ V}_{kn}\,\,the\,\,benefit\,\,criteria\,\,or\,\,Min\,\,of\,\,{ V}_{kn}\,\,the\,\,cost\,\,criteria\}$$

$${S}_{k}^{-}=\{Max{ V}_{kn}\,\,the\,\,cost\,\,criteria\,\,or\,\,Min\,\,of\,\,{ V}_{kn}\,\,the\,\,benefit\,\,criteria\}$$

##### In this step, the distance of each viable solution from both the ideal solution and the negative ideal solution is computed.

5 $${D}_{k}^{+}=\sqrt{\sum_{n=1}^{N}{\left({V}_{kn}-{S}_{k}^{+}\right)}^{2}}$$

6 $${D}_{k}^{-}=\sqrt{\sum_{n=1}^{N}{\left({V}_{kn}-{S}_{k}^{-}\right)}^{2}}$$

##### Computation of comparative degree of score estimate

Now, the relative closeness to the ideal solution is determined by the following Eq.

7 $${C}_{k}=\frac{{D}_{k}^{-}}{\left({D}_{k}^{+}+{D}_{k}^{-}\right)}, \left(0\le {C}_{k}\le 1;k=\mathrm{1,2},\dots .K\right)$$

Maximum value of ${C}_{k}$ indicates maximum rank.

The weights of both the main and sub-criteria were established using the SpiceLogic AHP Software, while all calculations employing the TOPSIS method were conducted in Microsoft Office Excel 2016 (MS Excel).

### Ethical approval

The regulations and guidelines that were applicable at the time of the plant trials used in this study were followed.

## Results and discussion

### Weight calculation of all main and sub-parameters using AHP

**Figure 3:**
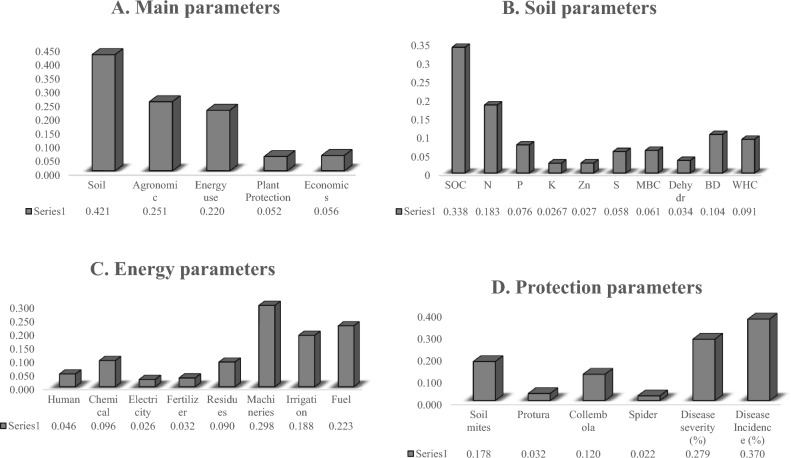
Presenting the weights of main (**A**), and sub-parameters of soil (**B**), energy (**C**) and protection (**D**).

The five main parameters—soil, agronomy, energy, plant protection, and economics as well as the related sub-parameters were compared comprehensively. The weights were determined (Tables 5, 6, 7, and 8) using the scores given by various experts in their respective disciplines (Supplemental material, S2–S5) following the Satty scoring chart (Table 4).The soil parameter had the highest weight when all the major parameters were weighted using the decision matrix (0.421), followed by agronomy (0.251), energy (0.220), economics (0.056), and plant protection (0.052) (Fig. 3A). Again, among the soil parameters, soil organic carbon (SOC) had the highest weight, followed by nitrogen (N), bulk density (BD), water holding capacity (WHC), phosphorus (P), microbial biomass carbon (MBC), sulfur (S), porosity, potassium (K), and zinc (Zn), in that order (Fig. 3B). Due to changes in the form of organic (residue) addition, the dynamics of nutrients in soils may change over those of chemically treated soils. Again, zero-tillage and residue addition may also have a substantial impact on plant nutrient availability due to such variations in the number of nutrients available and their distribution in the soil profile. Further, an increase in SOC due to the favorable three principles of CA increases the storage capacity of soils for N, P, and S and ensures better soil physical (WHC and BD) and microbiological status (MBC)^27,28^. The decision matrix of energy parameters (Fig. 3C) shows a maximum weight for machinery (0.298), followed by fuel (0.223), irrigation (0.188), chemical (0.096), residues (0.090), human (0.046), fertilizer (0.032), and electricity (0.026); while such weights of plant protection sub-parameters is maximum for disease incidence (0.370) followed by disease severity (0.279), soil mites (0. 178), Collembola (0.120), Protura (0.032), and spider (0.022) (Fig. 3D). The consistency ratio (CR) of the decision matrix of the main parameters is 5.2% (Table 5), while its CRs for soil, energy, and protection are 5.9%, 6.5%, and 9.5%, respectively (Tables 5, 6, 7, and 8). In fact, the CRs for all the above decision matrices are less than 10%, which indicates the presence of a sufficient consistency level for measuring the weights^29^.

**Table 5:** Decision Matrix for weights of all the five main parameters.

Main parameters	Soil	Agronomy	Energy	Plant protection	Economics
Soil	1	3	2	6	5
Agronomy	0.333	1	2	5	4
Energy	0.5	0.5	1	5	6
Plant protection	0.168	0.2	0.2	1	1
Economics	0.2	0.25	0.167	1	1
Weight	0.421	0.251	0.220	0.052	0.056

Consistency ratio (CR) = 5.2%, Principal eigenvalue = 5.232.

**Table 6:** Decision matrix of soil parameters.

Sub parameters	SOC	N	P	K	Zn	S	MBC	Dehyd	BD	WHC
SOC	1	4	7	8	8	6	4	7	4	4
N	0.250	1	4	7	6	4	3	7	2	2
P	0.143	0.25	1	4	3	2	1	6	0.333	0.5
K	0.125	0.143	0.25	1	1	0.5	0.333	1	0.333	0.333
Zn	0.125	0.167	0.333	1	1	0.333	0.5	1	0.333	0.25
S	0.167	0.25	0.5	2	3	1	2	3	0.333	0.333
MBC	0.25	0.333	1	3	2	0.5	1	2	0.5	1
Dehyd	0.143	0.143	0.167	1	1	0.333	0.5	1	0.5	1
BD	0.25	0.5	3	3	3	3	2	2	1	1
WHC	0.25	0.5	2	3	4	3	1	1	1	1
Weight	0.338	0.183	0.076	0.027	0.027	0.058	0.061	0.034	0.104	0.091

Consistency ratio (CR) = 5.9%, Principal eigenvalue = 10.23.

**Table 7:** Decision matrix of energy parameters.

Sub parameters	Human	Chemical	Electricity	Fertilizer	Residues	Machinery	Irrigation	Fuel
Human	1	0.5	3	2	0.333	0.167	0.25	0.167
Chemical	2	1	4	3	2	0.5	0.333	0.2
Electricity	0.333	0.25	1	0.5	0.2	0.125	0.333	0.125
Fertilizer	0.5	0.333	2	1	0.25	0.143	0.2	0.143
Residues	3	0.5	5	4	1	0.25	0.5	0.333
Machineries	6	2	8	7	4	1	3	2
Irrigation	4	3	3	5	2	0.333	1	2
Fuel	6	5	8	7	3	0.5	0.5	1
Weight (W_n_)	0.046	0.096	0.026	0.032	0.090	0.298	0.188	0.223

Consistency ratio (CR) = 6.5%, Principal eigenvalue = 8.63.

**Table 8:** Decision matrix of Plant protection parameters.

Sub parameters	Mites	Protura	Collembola	Spider	Disease severity (%)	Disease Incidence (%)
Soil mites	1	7	3	9	0.333	0.333
Protura	0.143	1	0.143	2	0.143	0.143
Collembola	0.333	7	1	9	0.5	0.143
Spider	0.111	0.5	0.111	1	0.111	0.111
Disease severity (%)	3	7	2	9	1	1
Disease Incidence (%)	3	7	7	9	1	1
Weight (W_n_)	0.178	0.032	0.120	0.022	0.279	0.370

Consistency ratio (CR) = 9.5%, Principal eigenvalue = 6.59.

**Figure 4:**
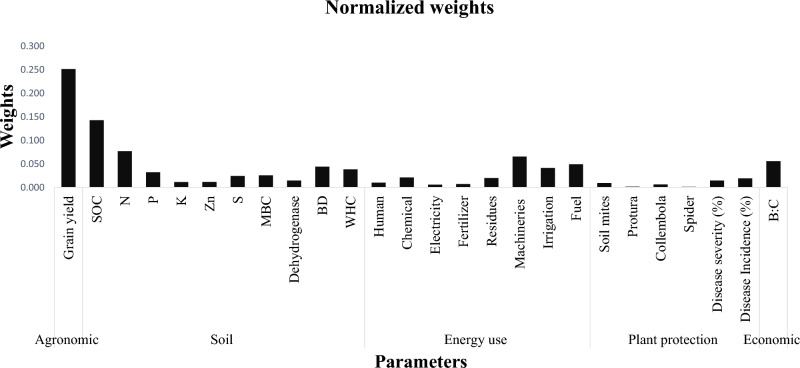
Showing normalized weights for all the sub-parameters considered.

Along with the CR values, the maximum eigenvalue of the decision matrix consisting of all the five main parameters is 5.232, which is nearer to 5 (Tables 5, 6, 7, and 8), the number of main parameters. Similarly, the maximum eigenvalue of the decision matrices for all the sub-parameters is 10.23 (soil), 8.63 (energy), and 6.59 (protection), which are almost equal to their corresponding number of sub-parameters (Tables 5, 6, 7, and 8). Consequently, the eigenvalues supported the constancy of the decision-preference makers throughout the examination. All the above CRs and eigenvalues thus have exhibited that the results are reliable. After giving the weights to all the five main parameters and corresponding sub-parameters, Fig. 4 is drawn with normalized weights of all the 23 sub-parameters under the main parameters.

### Ranking the alternatives

Using Eqs. (5)–(7), the distance from the ideal (${D}_{k}^{+}$), negative ideal ${(D}_{k}^{-})$, and relative proximity to the ideal solution ${(C}_{K})$ are calculated. The relative degree of approximation could be used to rank the options. The first ranked alternative for wheat cultivation was ZERO1 (0% residue + 100% NPK), followed by ZERO4 (50%residue + 100% NPK), ZERO2 **(**100% residue + 50% NPK), and CONV4 (50%residue + 100% NPK) (Table 9). Treatments receiving a higher crop residue in soil may cause an immobilization of nutrients, especially in the initialyears of CA implementation. This might lead to lower availability of nutrients in the soil. As the ZERO1 treatment received only the sole application of NPK, it performed superiorly in explaining an optimum plant nutrient availability in the TOPSIS method than the treatments receiving higher crop residue. A blending of crop residue (50/100%) with the inorganic application (100/50% NPK) under ZERO4 and ZERO2 treatments may also be promising ones where the inorganic application may replenish the nutrient availability gap created by immobilization and ranked second/third in this experiment.

**Table 9:** Showing rank of different alternatives based on TOPSIS method.

Alternatives	Ideal solution ($${D}_{k}^{+}$$)	Negative solution ($${D}_{k}^{-})$$	Relative closeness ($${C}_{k})$$	(Rank)
CONV1	0.097	0.093	0.488	4
CONV2	0.118	0.058	0.328	14
CONV3	0.131	0.073	0.358	13
CONV4	0.115	0.069	0.375	12
CONV5	0.135	0.056	0.295	15
REDUC1	0.116	0.086	0.427	10
REDUC2	0.115	0.083	0.418	11
REDUC3	0.109	0.084	0.434	8
REDUC4	0.111	0.084	0.430	9
REDUC5	0.105	0.092	0.467	7
ZERO1	0.091	0.106	0.538	1
ZERO2	0.101	0.099	0.497	3
ZERO3	0.103	0.097	0.487	5
ZERO4	0.090	0.094	0.511	2
ZERO5	0.095	0.087	0.476	6

In rice–wheat cropping system, moving from a conventional system to an intensified CA-based system can reduce production costs while maintaining or improving yields, soil health, and profitability. While conservation agricultural practices (zero tillage) increase biodiversity and soil fertility, conventional tillage or intense tillage operations lower the population of soil mesofauna (soil mites, proturan, collembola, and soil spider) and their diversity in soil. When compared to traditional tillage, the use of zero tillage can also considerably lower the population of certain soil-borne plant pathogenic fungi^30^. Therefore, the cheap cost of tillage operation and improved energy usage efficiency were responsible for the good performance of wheat crop under zero tillage. All these factors collectively enhance the economic profitability of zero tillage in comparison to alternatives like reduced and conventional tillage. Similar research claimed the total energy input was at its lowest in zero tillage with no residue retention (74,688 MJ ha^−1^) and at its highest in conservation tillage with 100% residue retention (150,392 MJ ha^−1^)^31^.

### Discussion

Conservation agriculture ensures ecological and economic sustainability of a production system through less tillage, more soil cover, and improved rotations. It thus requires more sophisticated decision-making. While it is vital to consider many criteria simultaneously, the varying degrees of influence these criteria have on adopting conservation management measures may introduce contradictory goals.

Integrating AHP and TOPSIS methods, we obtained a comparable performance in our study. One of the challenges of combining both the methods is to identify the relative importance or weight of the criteria used in the process of decision making. The AHP approach was practical in using data with multiple and subjective knowledge, and it determined the weights of several criteria and sub-criteria while measuring any inconsistency in assessment. However, each method has significant limitations. For example, AHP, relying on subjective emotions and potential difficulties in assigning numerical characteristics, and TOPSIS, focusing on Euclidean distance while ignoring attribute correlation, exhibit notable limitations in their respective approaches^32^.

The AHP method addresses a common problem during pairwise comparisons: expert judgments may make assigning numerical values difficult due to their influence by human emotions, and the technique effectively confirms logical consistency in the comparisons. Use of both the methods may thus give robustness and comprehensiveness for selecting the best alternatives.

The consistency ratios (Tables 5, 6, 7, and 8) in this study were less than 10%, indicating that tolerable inconsistencies exist, allowing for the progression of the analysis. Similarly, the maximum eigenvalue of the decision matrix, encompassing all primary parameters, closely approximates 5 (5.232), corresponding to the number of primary parameters. Along with the main parameters, the sub-parameters have also shown similar results (Tables 5, 6, 7, and 8). The eigenvalues thus confirmed the stability of decision-makers preference throughout the investigation^29,32–34^. TOPSIS determines the ranking of the [assessment]alternatives by calculating the difference between ideal and negative ideal solutions. It is remarkably versatile and relevant to our present investigation, since it maximizes the utilization of the original data without placing strict constraints on the quantity of data samples.

The Shannon entropy employs a discrete probability distribution to measure the uncertainties inherent in the information source^35^; while Chen^36^ reported that entropy weight might lead to unreasonable decision-making or evaluation results. Seyedmohammadi et al.^37^ employed a fuzzy analytic network process to calculate the weight of criteria in studying the land suitability potential in agriculture. However, Li et al.^38^ noted that a relatively large sample size is necessary when employing the fuzzy evaluation method for sustainability assessment. Considering existence of all these opinions, we employed AHP for determining the weights assigned to the criteria in our study, since previous research (Zoma and Sawadogo^34^, Adhikari et al.^39^ and Abdulvahitoglu and Kilic^40^) endorsed such use. Subsequently, we applied these criteria weights to TOPSIS to rank the alternatives; and it (TOPSIS) demonstrated that zero tillage outperformed conventional and reduced tillage (Table 9), using ratings based on relative closeness to the ideal solution (C_k_). Such superiority of zero tillage under rice–wheat cropping system was also reported earlier^3,41,42^. Therefore, the alternative ranking produced from the TOPSIS technique is formally recognized. According to the results of the previous study, integrating TOPSIS with AHP may thus be a good tool for a fair ranking of the alternatives to find out the best.

The MCDM approach effectively addresses incompatibility issues. Despite some research on MCDM in agriculture, particularly AHP-TOPSIS, more investigation is needed due to varied approaches in decision-making scenarios. Agriculture decision-making is complex, like in other sectors, and determining criteria weights is a crucial first step.

Our results thus clearly show that the methods presented may help agriculture decision-makers to identify optimal management practices for CA with rice–wheat cropping systems in the eastern Indo-Gangetic plains in particular. The AHP-TOPSIS model, unlike traditional methods, considers the decision-makers preferences and conducts a regional-scale assessment, evaluating criteria and sub-criteria separately, which allows for a nuanced understanding of factors like soil quality, grain yield, plant protection, energy use, and economic considerations. Including more data on agronomy, physiology and genetics can enhance the efficiency of the methods.

## Conclusions

The combined model of AHP and TOPSIS provides a useful tool for examining appropriate CA practices. Multiple criteria are used to analyze the problems, and the results are ranked and arranged in a systematic framework. The relative weightage of all the criteria of five disciplines was measured using the AHP methods, while TOPSIS ranked the performance of different alternatives based on the weightage in various criteria. Of the 15 alternatives tested, performance of ZERO1 (0% residue + 100% NPK) was found the best, followed by ZERO4 (50% residue + 100% NPK) and ZERO2 **(**100% residue + 50% NPK) for upscaling CA with wheat in rice–wheat system for lower Indo-Gangetic plains. However, testing consistency of the model (AHP-TOPSIS) with CA over long periods and in various agroecological zones is crucial.

### Supplementary Information

Supplementary Information.

## Data Availability

The datasets utilized in the present study are not accessible to the public as per the approved experimental protocol. However, interested individuals can obtain them by making a reasonable request to the corresponding author.
