# Supplementary material for: Evaluation of management practices in rice–wheat cropping system using multicriteria decision-making methods in conservation agriculture
Source: Sci Rep. 2024 Apr 13;14:8600. doi: 10.1038/s41598-024-58022-w (PMC11016099; doi:10.1038/s41598-024-58022-w)
Supplement: Supplementary file 1 — Supplementary Information. [file 41598_2024_58022_MOESM1_ESM.pdf]

Evaluation of Management Practices in Rice-Wheat Cropping System Using Multicriteria Decision Making Methods in Conservation Agriculture

TufleuddinBiswas[1,2*], Anurup Majumder[2], Shamik Dey[3], Anwesha Mandal[4], Soumik Ray[1], Promil Kapoor[5], Walid Emam[6], Sahely Kanthal[7], Alessio ISHIZAKA[8], Adelajda Matuka[9]

'Authors' affiliation:

[1]*Department of Agricultural Economics and Statistics, Centurion University of Technology and Management (CUTM), Odisha-761211, India*

[2]*Department of Agricultural Statistics, Bidhan Chandra Krishi Viswavidyalaya, West Bengal-741252, India*

[3]*School of Agricultural Sciences, JIS University, West Bengal-700109, India*

[4]*Department of Agriculture Extension, G D Goenka University, Haryana, India*

[5]*Assistant Scientist, Plant Pathology, CCSHAU, Hisar, India*

[6]*Faculty of Science, King Saud University, P.O. Box 2455, Riyadh 11451, Saudi Arabia*

[7]*School of Agriculture, Swami Vivekananda University, Barrackpure, West Bengal 700121*

[8]*Department of Supply Chain and Decision Making, NEOMA Business School, Rouen, France*

[9]*Department of Economics, University of Bologna, 40126 Bologna, Italy*

***Corresponding Author-** *Email:* tufleuddinbiswas@gmail.com

Supplementary Material

S1. List of the Experts whose ratings were used to determine the weights of the various main and sub-parameters wheat crop

Sl. No	Parameters	Name and designation of the experts
1	Main Parameters (Table: S2)	I. Prof. Biswapati Mandal, Professor, Department of Agricultural Chemistry and Soil Science<br>II. Prof. Mahadev Pramanick, Professor & Head, Department of Agronomy<br>III. Prof. Krishna Karmakar, Professor , Department of Agricultural Entomology<br>IV. Prof. Srikanta Das, Professor, Department of Plant Pathology<br>V. Dr. Soumitra Chatterjee Assistant Professor, Department of Agricultural Economics
2	Soil Parameters (Table-S3)	I. Prof. Biswapati Mandal, Professor, Agricultural Chemistry & Soil Science<br>II. Dr. Dibyendu Sarkar, Assistant Professor, Agricultural Chemistry & Soil Science<br>III. Dr. Kaushik Batobyal, Assistant Professor, Agricultural Chemistry & Soil Science<br>IV. Dr. Sidhu Murmu, Assistant Professor, Agricultural Chemistry & Soil Science
3	Energy (Table-S5)	Dr. Subrata Karmakar, Professor & Head, Department of Farm Machinery & Power
4.	Crop Protections (Table-S4)	I. Prof. Krishna Karmakar, Professor , Department of Agricultural Entomology<br>II. Prof. Srikanta Das, Professor, Department of Plant Pathology<br>III. Dr. Subrata Dutta, Assistant Professor, Department of Plant Pathology

QUESTIONNAIRE FOR THE CASE STUDY DEVELOPMENT
Circle one number per row below using the scale:

1 = Equal 3 = Moderate 5 = Strong 7 = Very strong 9 = Extreme
2, 4, 6, 8 are intermediate values

S2. Compare the relative performance of one criterion against **all other selection criteria** for the **Selection of best tillage alternative (s) for wheat crop under CA**.

Soil	9	8	7	**6**	5	4	3	2	1	2	3	4	5	6	7	8	9	Agronomy
Soil	9	8	7	6	5	**4**	3	2	1	2	3	4	5	6	7	8	9	Energy
Soil	9	8	**7**	6	5	4	3	2	1	2	3	4	5	6	7	8	9	Protection
Soil	9	8	**7**	6	5	4	3	2	1	2	3	4	5	6	7	8	9	Economics
Agronomy	9	8	7	6	5	4	3	2	**1**	2	3	4	5	6	7	8	9	Energy
Agronomy	9	8	7	6	**5**	4	3	2	1	2	3	4	5	6	7	8	9	Protection
Agronomy	9	8	7	6	5	**4**	3	2	1	2	3	4	5	6	7	8	9	Economics
Energy	9	8	7	6	**5**	4	3	2	1	2	3	4	5	6	7	8	9	Protection
Energy	9	8	7	**6**	5	4	3	2	1	2	3	4	5	6	7	8	9	Economics
Protection	9	8	7	6	5	4	3	2	**1**	2	3	4	5	6	7	8	9	Economics

S3. Compare the relative performance of one soil sub criteria against **all other soil sub criteria** for the selecting **best tillage alternative (s) for Wheat crop under CA.**

SOC	9	8	7	6	5	**4**	3	2	1	2	3	4	5	6	7	8	9	N
SOC	9	8	**7**	6	5	4	3	2	1	2	3	4	5	6	7	8	9	P
SOC	9	**8**	7	6	5	4	3	2	1	2	3	4	5	6	7	8	9	K
SOC	9	**8**	7	6	5	4	3	2	1	2	3	4	5	6	7	8	9	Zn
SOC	9	8	7	**6**	5	4	3	2	1	2	3	4	5	6	7	8	9	S
SOC	9	8	7	6	5	**4**	3	2	1	2	3	4	5	6	7	8	9	MBC
SOC	9	8	**7**	6	5	4	3	2	1	2	3	4	5	6	7	8	9	DHA
SOC	9	8	7	6	5	**4**	3	2	1	2	3	4	5	6	7	8	9	BD
SOC	9	8	7	6	5	**4**	3	2	1	2	3	4	5	6	7	8	9	WHC
N	9	8	7	6	5	**4**	3	2	1	2	3	4	5	6	7	8	9	P
N	9	8	**7**	6	5	4	3	2	1	2	3	4	5	6	7	8	9	K
N	9	8	7	**6**	5	4	3	2	1	2	3	4	5	6	7	8	9	Zn
N	9	8	7	6	5	**4**	3	2	1	2	3	4	5	6	7	8	9	S
N	9	8	7	6	5	4	**3**	2	1	2	3	4	5	6	7	8	9	MBC
N	9	8	**7**	6	5	4	3	2	1	2	3	4	5	6	7	8	9	DHA
N	9	8	7	6	5	4	3	**2**	1	2	3	4	5	6	7	8	9	BD
N	9	8	7	6	5	4	3	**2**	1	2	3	4	5	6	7	8	9	WHC
P	9	8	7	6	5	**4**	3	2	1	2	3	4	5	6	7	8	9	K
P	9	8	7	6	5	4	**3**	2	1	2	3	4	5	6	7	8	9	Zn
P	9	8	7	6	5	4	3	**2**	1	2	3	4	5	6	7	8	9	S
P	9	8	7	6	5	4	3	2	**1**	2	3	4	5	6	7	8	9	MBC
P	9	8	7	**6**	5	4	3	2	1	2	3	4	5	6	7	8	9	DHA
P	9	8	7	6	5	4	3	2	1	2	**3**	4	5	6	7	8	9	BD
P	9	8	7	6	5	4	3	2	1	**2**	3	4	5	6	7	8	9	WHC
K	9	8	7	6	5	4	3	2	**1**	2	3	4	5	6	7	8	9	Zn
K	9	8	7	6	5	4	3	2	1	**2**	3	4	5	6	7	8	9	S
K	9	8	7	6	5	4	3	2	1	2	**3**	4	5	6	7	8	9	MBC
K	9	8	7	6	5	4	3	2	**1**	2	3	4	5	6	7	8	9	DHA
K	9	8	7	6	5	4	3	2	1	2	**3**	4	5	6	7	8	9	BD
K	9	8	7	6	5	4	3	2	1	2	**3**	4	5	6	7	8	9	WHC
Zn	9	8	7	6	5	4	3	2	1	2	**3**	4	5	6	7	8	9	S
Zn	9	8	7	6	5	4	3	2	1	**2**	3	4	5	6	7	8	9	MBC
Zn	9	8	7	6	5	4	3	2	**1**	2	3	4	5	6	7	8	9	DHA
Zn	9	8	7	6	5	4	3	2	1	2	**3**	4	5	6	7	8	9	BD
Zn	9	8	7	6	5	4	3	2	1	2	3	**4**	5	6	7	8	9	WHC
S	9	8	7	6	5	4	3	**2**	1	2	3	4	5	6	7	8	9	MBC

S	9	8	7	6	5	4	**3**	2	1	2	3	4	5	6	7	8	9	DHA
S	9	8	7	6	5	4	3	2	1	2	**3**	4	5	6	7	8	9	BD
S	9	8	7	6	5	4	3	2	1	2	**3**	4	5	6	7	8	9	WHC
MBC	9	8	7	6	5	4	3	**2**	1	2	3	4	5	6	7	8	9	DHA
MBC	9	8	7	6	5	4	3	2	1	**2**	3	4	5	6	7	8	9	BD
MBC	9	8	7	6	5	4	3	2	**1**	2	3	4	5	6	7	8	9	WHC
DHA	9	8	7	6	5	4	3	2	**1**	2	3	4	5	6	7	8	9	WHC
BD	9	8	7	6	5	4	3	2	**1**	2	3	4	5	6	7	8	9	WHC

BD-Bulk density, WHC-Water holding capacity, SOC- soil organic C, N-Nitrogen, P-Phosphorus, K-Potassium, Zn-Zinc, S-Sulphur, MBC-Microbial biomass carbon, DHA- Dehydrogenase activity

S4. Compare the relative performance of one protection sub criterion against **all other plant protection sub criteria** for the selecting **best tillage alternative (s) for Wheat crop under CA**.

Soil Mites	9	8	7	6	5	4	3	2	1	2	3	4	5	6	7	8	9	Protura
Soil Mites	9	8	7	6	5	4	3	2	1	2	3	4	5	6	7	8	9	Collembola
Soil Mites	9	8	7	6	5	4	3	2	1	2	3	4	5	6	7	8	9	Spider
Soil Mites	9	8	7	6	5	4	3	2	1	2	3	4	5	6	7	8	9	Disease severity
Soil mites	9	8	7	6	5	4	3	2	1	2	3	4	5	6	7	8	9	Disease incidence
Protura	9	8	7	6	5	4	3	2	1	2	3	4	5	6	7	8	9	Collembola
Protura	9	8	7	6	5	4	3	2	1	2	3	4	5	6	7	8	9	Spider
Protura	9	8	7	6	5	4	3	2	1	2	3	4	5	6	7	8	9	Disease severity
Protura	9	8	7	6	5	4	3	2	1	2	3	4	5	6	7	8	9	Disease incidence
Collembola	9	8	7	6	5	4	3	2	1	2	3	4	5	6	7	8	9	Spider
Collembola	9	8	7	6	5	4	3	2	1	2	3	4	5	6	7	8	9	Disease severity
Collembola	9	8	7	6	5	4	3	2	1	2	3	4	5	6	7	8	9	Disease incidence
Spider	9	8	7	6	5	4	3	2	1	2	3	4	5	6	7	8	9	Disease severity
spider	9	8	7	6	5	4	3	2	1	2	3	4	5	6	7	8	9	Disease incidence
Disease severity	9	8	7	6	5	4	3	2	1	2	3	4	5	6	7	8	9	Disease incidence

5. Compare the relative performance of one Engineering sub criteria against **all other Engineering (energy) sub criteria** for the selecting **best tillage alternative (s) for Wheat crop under CA.**

Human Labour	9	8	7	6	5	4	3	2	1	2	3	4	5	6	7	8	9	Plant Protection Chemical
Human Labour	9	8	7	6	5	4	3	2	1	2	3	4	5	6	7	8	9	Electricity
Human Labour	9	8	7	6	5	4	3	2	1	2	3	4	5	6	7	8	9	Inorganic Fertilizer
Human Labour	9	8	7	6	5	4	3	2	1	2	3	4	5	6	7	8	9	Residue
Human Labour	9	8	7	6	5	4	3	2	1	2	3	4	5	6	7	8	9	Machineries
Human Labour	9	8	7	6	5	4	3	2	1	2	3	4	5	6	7	8	9	Irrigation
Human Labour	9	8	7	6	5	4	3	2	1	2	3	4	5	6	7	8	9	Fuel Consumption
Plant Protection Chemical	9	8	7	6	5	4	3	2	1	2	3	4	5	6	7	8	9	Electricity
Plant Protection Chemical	9	8	7	6	5	4	3	2	1	2	3	4	5	6	7	8	9	Inorganic Fertilizer
Plant Protection Chemical	9	8	7	6	5	4	3	2	1	2	3	4	5	6	7	8	9	Residue
Plant Protection Chemical	9	8	7	6	5	4	3	2	1	2	3	4	5	6	7	8	9	Machineries
Plant Protection Chemical	9	8	7	6	5	4	3	2	1	2	3	4	5	6	7	8	9	Irrigation
Plant Protection Chemical	9	8	7	6	5	4	3	2	1	2	3	4	5	6	7	8	9	Fuel Consumption
Electricity	9	8	7	6	5	4	3	2	1	2	3	4	5	6	7	8	9	Inorganic Fertilizer
Electricity	9	8	7	6	5	4	3	2	1	2	3	4	5	6	7	8	9	Residue

Electricity	9	8	7	6	5	4	3	2	1	2	3	4	5	6	7	**8**	9	Machineries
Electricity	9	8	7	6	5	4	3	2	1	2	**3**	4	5	6	7	8	9	Irrigation
Electricity	9	8	7	6	5	4	3	2	1	2	3	4	5	6	7	**8**	9	Fuel Consumption
Inorganic Fertilizer	9	8	7	6	5	4	3	2	1	2	3	**4**	5	6	7	8	9	Residue
Inorganic Fertilizer	9	8	7	6	5	4	3	2	1	2	3	4	5	6	**7**	8	9	Machineries
Inorganic Fertilizer	9	8	7	6	5	4	3	2	1	2	3	4	**5**	6	7	8	9	Irrigation
Inorganic Fertilizer	9	8	7	6	5	4	3	2	1	2	3	4	5	6	**7**	8	9	Fuel Consumption
Residue	9	8	7	6	5	4	3	2	1	2	3	4	5	6	7	8	9	Machineries
Residue	9	8	7	6	5	4	3	2	1	**2**	3	4	5	6	7	8	9	Irrigation
Residue	9	8	7	6	5	4	3	2	1	2	**3**	4	5	6	7	8	9	Fuel Consumption
Machineries	9	8	7	6	5	4	**3**	2	1	2	3	4	5	6	7	8	9	Irrigation
Machineries	9	8	7	6	5	4	3	**2**	1	2	3	4	5	6	7	8	9	Fuel Consumption
Irrigation	9	8	7	6	5	4	3	2	1	2	**3**	4	5	6	7	8	9	Fuel Consumption
